# Supplementary material for: Are carbon emissions trading and green financial instruments synergistic? -Comprehensive quantitative research based on content analysis
Source: PLoS One. 2024 Mar 7;19(3):e0298601. doi: 10.1371/journal.pone.0298601 (PMC10919601; doi:10.1371/journal.pone.0298601)
Supplement: S1 Data — (ZIP) [file pone.0298601.s001.zip › Data/地级市Z_F工作报告.zip!/╟α║ú/╬≈─■╩╨/└┤╫╘╣1⁄2╓┌║┼í╛┬φ┐╦╩2╛▌═°í┐_2013.docx]

各位代表：

现在，我代表市人民政府向大会作工作报告，请予审议，并请市政协各位委员和列席会议的同志提出意见。

一、2012年工作回顾

2012年，在省委、省政府和市委的坚强领导下，我们团结带领和紧紧依靠全市各族干部群众，以科学发展观为指导，深入贯彻落实党的十八大精神，按照省第十二次党代会提出的"两新"目标和"三区"战略任务，围绕建设"生活之城、幸福之城"的奋斗目标，着力稳增长、调结构，抓改革、促发展，控物价、惠民生，全面完成了市十五届人大二次会议确定的各项目标任务，取得了经济社会持续快速发展、城乡面貌显著变化、生态环境明显改善、人民生活水平不断提高的新成就。全年实现地区生产总值851.09亿元，增长15%；实现公共财政预算收入122.7亿元，增长20.4%；总财力达到192.6亿元，增长23.5%；地方公共财政预算收入达到54.8亿元，增长21%；财政支出185.3亿元，增长23.5%；完成固定资产投资700.5亿元，增长32.7%；完成社会消费品零售总额317.5亿元，增长17%；城镇居民人均可支配收入17633元，增长11.3%；农民人均纯收入7801元，增长17.6%。地区生产总值、公共财政预算收入、社会消费品零售总额等指标增幅分别高于全省2.1、2.2和1.4个百分点。其中，生产总值增幅高于全国7.2个百分点，发展的质量和效益显著提升。

　　一年来，重点做了以下八个方面的工作：

　　（一）突出项目建设，投资拉动作用显著。以"项目推进年"为有力支撑，项目工作实现重大突破。落实了城市供排水管网改造、循环经济等一批项目专项资金，实施各类建设项目809个。"畅通交通"工程等一批重点项目加快推进，完成火车站综合改造、教育布局调整等重点项目年度建设任务。电子铝箔、锂电材料等重点工业项目建设进展顺利，一批新项目实现投产。坚持以融资促项目，全年融资额达100多亿元，融资工作实现重大突破。西宁环境综合治理项目列入世界银行贷款规划；分别与国家开发银行和太平洋建设集团签订了500亿元开发合作项目和120亿元的基础设施建设合作框架意向协议，落实了39亿元火车站综合改造贷款。西宁城投与国开金融合作，设立了西宁城市投资发展基金。全年实现民间投资449.8亿元,占全部投资的64.2%。完成项目拆迁512万平方米、拆除违章建筑58万平方米，拆迁是前五年拆迁量之和，征地拆迁工作实现重大突破，有力保障了重大项目顺利实施！

　　（二）优化产业结构，发展方式加快转变。认真落实省政府应对工业经济突出问题的十项举措和支持小微企业发展的六十条政策，扎实开展"帮企业、稳增长、调结构"活动，加快特色优势产业发展和循环经济产业培育，积极推进小微企业发展，成立了小微企业服务中心。民营经济发展势头良好，私营企业达到12162户，注册资金258.3亿元；个体工商户62614户，注册资金20.4亿元。年内新增各类企业2653家，培育市场主体实现重大突破。加强与金融机构沟通协调，帮助企业争取流动资金贷款30多亿元、专项扶持资金9.7亿元，缓解了企业资金困难。全年完成工业增加值377.2亿元，增长19.5%；其中，规模以上工业增加值323.1亿元，增长15.1%。加大为园区发展服务的力度，筹资2亿元支持基础设施建设。园区实现工业增加值227.5亿元，增长27.4%，占全市工业增加值比重达到60.7%。促进文化旅游产业融合发展，大力扶持发展小微宾馆，加快培育消费市场。西宁再度入选"中国十大旅游避暑城市"，成功创建塔尔寺国家5A级景区，大通县荣膺"2012中国十佳城市慢游地"称号。全年共接待国内外游客1127.6万人次，同比增长14.9%；实现旅游收入75.2亿元，增长33.8%。加快商贸、餐饮等传统服务业改造升级和物流、信息等现代服务业发展，全年完成三产增加值380.4亿元，增长11.8%。加快科技创新，实施重大科研项目42项，创新型城市建设稳步推进。

　　（三）统筹城乡发展，推进"三农"工作。强化各项支农惠农政策，大力推进农业发展，"大通模式"受到国家有关部门肯定。全市农作物播种面积达183.2万亩，特色优势农作物占总播种面积的75%以上。完成一产增加值31.17亿元，增长5.3%。狠抓设施农业，完成冬暖式温棚3100栋、畜棚4000栋，新建各类养殖基地55家。实施75个贫困村整村推进和17个村易地搬迁项目，完成农村人畜饮水工程17项，实现了减少5万贫困人口的目标。组织引导农村劳动力转移就业37.7万人次，实现劳务收入17.5亿元。投资13.7亿元，完成了111个"党政军企共建示范村"建设任务，15.9万农民群众从中受益，城乡统筹发展迈出新的步伐！

　　（四）坚持规划先行，城市面貌日新月异。牢固树立规划先行理念，实行全域规划，规划工作实现重大突破。开展《西宁都市区2030年战略规划》编制及《2020西宁市总体规划》修改工作，完成了三县总体规划编制，实现控制性详规全覆盖目标。以提升城市特色、内涵和品质为出发点，编制了20余处重点片区规划和《城市色彩专项规划》、《城市夜景总体规划》及给水工程、核心城区路网优化等专项规划。认真进行重大项目建设前期规划论证，落实了区域用地规划布局，规划建设城市的科学化水平进一步提升。在规划引领下，旧城改造、保障性住房建设、生态环境治理和一批公共服务设施工程加快推进，城市面貌发生显著变化。

　　（五）注重生态环境，宜居质量持续改善。完成大南山生态绿色屏障2.68万亩人工造林和74个新农村村庄绿化，投资5.7亿元完成各项林业生态建设26.3万亩，义务植树490万株，全市森林覆盖率达到29.6%。新增园林绿地40公顷，人均公共绿地面积达到10平方米。京韵青风景区、朝阳绿岛等主题公园建成使用。扎实推进"创模"工作，实施减排项目30项。开展"煤改气"和餐饮业煤（油）烟污染专项治理工作，全年空气质量优良率达到86%。列入全国示范工程的大通县煤矿地质环境治理项目全面启动，提前一年完成了历史遗留铬渣处置任务，"两废"中心项目建设进展顺利。北川河核心段、南川河综合治理等"清水入城"工程加快推进，关停非法采洗砂场209处，整治排水管道19公里，排污口98个，开工建设2个污水处理厂，城市生活污水集中处理率达到90%，辖区河流水质全面实现了省政府要求的阶段性工作目标。餐厨垃圾处理"西宁模式"进一步深化，收运体系覆盖三县，处理率达到90%。生活垃圾无害化处理率达到92.3%，居全国前列。筹资9228万元实施了城市夜景亮化。投资9665万元实施了129个农村环境连片整治示范项目，城乡生态环境不断得到改善，宜居水平进一步提高！

　　（六）倾心社会民生，幸福指数不断提升。高度重视民生福祉，财政用于民生方面支出达80.6%。认真落实各项就业再就业政策，城镇新增就业2.8万人，各类失业人员再就业2.1万人，登记失业率为3.47%，创业带动就业工作受到国务院表彰。新型农村社会养老保险参保率达到94%，城镇居民社会养老保险新增20250人，均超额完成目标任务。社会保障体制机制进一步完善。实施了殡葬惠民政策。新开工建设城镇保障性住房14050套，竣工18474套，入住17424套；建设农村奖励性住房17000户，农村困难群众危房改造7479户。康川新城安置小区基本建成，2690户搬迁户享受到城市生活。完成教育重点工程投资9.6亿元，实施校安工程重点建设项目49个，完成8个农村初中校舍和25.05万平方米D级危房年度改造任务，全市73.06万平方米D级危房改造三年任务全面完成。教育布局调整一期13个项目顺利推进，二期调整17个项目启动实施。注重教育内涵发展，着力抓管理、提质量、保安全，教育均衡、协调、可持续发展迈出坚实步伐。不断扩大基本医保覆盖面，全市职工参保率达到99%，城镇居民参保率达到97%，新农合参保率达到97.8%，城乡居民医保人均筹资标准和住院报销比例均有提高。投资近4亿元，实施了急救中心、乡镇卫生院、社区及村级医疗服务机构等项目。在市、县级公立医院全面实行"先住院后结算"，公立医疗机构全部取消药物加成，在全国首创了床旁结算服务模式。运用商业保险解决城乡居民大病医疗保障走在全国前列，医改红利正在惠及城乡群众。推进基本公共服务向流动人口拓展和延伸，初步建立了流动人口计划生育均等化服务体系。大力发展文化事业和文化产业，青海大剧院建成并与中演公司合作运营，一批国内外高水平剧目进入我市。2012中国&middot;青海西宁国际原生态舞蹈暨现代舞艺术节、中国&middot;西宁FIRST青年电影节成功举办，"百姓大舞台"等文化体育活动日益丰富。持续推进"畅通交通"工程，新建、续建天桥22座，建成跨南川河3座步行桥，完成8个路口改造，开通4条快速公交专线，新购80辆、更新407辆公交车，市民出行更加安全舒适快捷。提高环卫工人工资，改善了工作和生活环境。采取多种措施稳控物价，全市居民消费价格累计涨幅为2.7%。年初确定的47项民生工程办实事项目顺利完成，全面兑现了向全市人民的郑重承诺！

　　（七）推进改革开放，精细管理全面推行。全面落实精细化工作要求，精细化管理实现重大突破。创新城市精细化管理模式，实施"街道长"和"楼院长"工作制度。全力推进全国文明城市创建，开展社会管理创新综合试点工作，推行"网格化管理、心连心服务"综治工作新模式。积极推进国库集中支付和非税收入收缴管理改革，全面启动区（县）公务卡改革，公开了市级"三公"经费预（决）算。深入推进文化体制改革，荣获"全国文化体制改革工作先进地区"称号。户籍制度改革试点取得突破性进展。同国内外交流合作有新成效，服务"青洽会"、成功举办首届"城洽会"，共签约落地项目101个。全年引进资金487亿元，招商引资工作实现重大突破！

　　（八）加强民主法治，政府效能不断提高。努力优化政务环境，提高行政效能，政府决策更加科学、民主、透明，为民、务实、廉洁、高效的要求进一步落实。扎实推进依法治市，各级政府自觉接受人大、政协的法律监督和民主监督，加强与人大代表、政协委员及各民主党派和社会各界人士的联系，认真办理市人大代表意见建议和市政协委员提案，办复率均为100%，代表、委员的满意率继续提升。高度重视人民来信来访，有效化解社会矛盾。加快"平安西宁"建设，依法打击各类违法犯罪活动。全力做好维稳工作，严厉打击邪教组织和活动。加强安全生产和食品药品监管。认真落实党的民族宗教政策，扎实推进"民族团结进步示范区"建设，民族团结、社会和谐稳定的政治局面进一步巩固和发展。审计、监察、工商、统计、质监等工作取得新进步，妇女儿童、残疾人等事业有了新发展。

　　玉树援建扎实推进，60个援建项目累计完成投资10.9亿元，占计划投资的93.4%，基本完成了援建任务，做出了省会城市应有的贡献。

　　回顾一年来的工作，成绩来之不易，这是省委、省政府和市委坚强领导的结果，是全市各族人民艰苦奋斗与开拓进取的结果，是市人大及其常委会依法有效监督和市政协民主监督、鼎力支持的结果。在此，我代表市人民政府，向各位人大代表、政协委员，向各民主党派、工商联、无党派人士和各人民团体，向奋斗在全市各条战线、各行各业的建设者和劳动者，向中央驻宁单位、省直各部门、人民解放军驻宁部队指战员、武警官兵和公安干警，向所有关心、支持西宁建设和发展的各界人士，致以崇高的敬意和衷心的感谢！

在肯定成绩的同时，我们也清醒地看到，受国际国内经济环境的影响，全市经济发展的不确定因素明显增多，还面临诸多的困难与挑战。经济整体实力还不强，创新驱动能力不足，城镇化发展不均衡，公共服务水平较低，稳控物价的任务依然艰巨，经济社会发展和政府行政能力与人民群众的新要求、新期盼还有一定差距。对各种困难和问题，我们将不回避、不畏难，以勇于负责、敢于担当的精神，采取有效措施，认真加以克服和解决。

二、2013年总体目标和主要任务

　　今年是全面深入贯彻落实党的十八大精神的开局之年，做好各项工作意义重大。今年政府工作的指导思想是：全面贯彻落实党的十八大、中央经济工作会议和省委十二届二次、三次全体会议和市委十三届六次全体会议精神，以科学发展观为指导，围绕省委"两新"目标、"三区"战略任务，抓住加快发展和转变方式两大关键，不断深化市情认识，不断强化精细工作理念，深入实施"一统三基"战略和"五个三"工程，注重投资拉动和产业支撑，加快优化需求结构、产业结构和区域结构，突出城市科学规划建设，突出产业做大做强，突出文化旅游体育融合发展，突出生态文明建设，突出惠民生控物价，全力攻坚文明城市创建，抓住重点、突破难点、体现特点、干出亮点，在全面建成小康社会上迈出新步伐，努力建设人民满意的"生活之城、幸福之城"。

　　党的十八大提出了2020年全面建成小康社会的战略目标。据测算，我市小康社会2011年实现程度为74.3%，相当于全国2008年的平均水平。确保与全国同步全面建成小康社会，要求我们在未来八年里，年复一年更加努力奋斗。今年经济社会发展的主要预期目标是：地区生产总值增长14%；地方公共财政预算收入增长17%；固定资产投资增长20%；社会消费品零售总额增长16%；旅游总收入增长22%；城镇居民人均可支配收入增长11%，农民人均纯收入增长14%；居民消费价格涨幅控制在5%以内；城镇登记失业率控制在4%以内；节能减排控制在省下达目标以内。

　　各位代表，做好今年各项工作，我们必须始终保持攻坚克难的奋斗姿态，始终保持尊重规律的求是态度，始终保持奉献人民的实干作风，始终保持精细管理的工作追求。精细管理是现阶段提升政府行政能力的重要抓手，体现奋斗、求是、务实，有很强的针对性、现实性、创新性，要坚持不懈地推行，积极有效地探索。要界定职权、责任，努力做到政府职权法授、程序法定、行为法限、责任法究。按照标准化、流程化、协同化、精细化的要求，强化精细工作理念，完善精细工作机制，提升精细工作效率，在城市规划建设、产业发展提升、民生保障改善、社会管理创新、项目组织管理、资金管理使用等各方面全面深化精细管理，让人民群众不断感受精细管理与服务带来的深刻变化！

　　在具体工作中，我们将力争在七个方面实现新的突破。

　　（一）全力以赴抓好项目建设，在扩大投资上实现新突破

　　投资对促进经济增长直接而有效，是工业化、城镇化和农业现代化的有力支撑。今年，全力抓好654个重点项目，努力实现840亿元以上的投资规模，保持20%以上的增长速度。

做好项目前期工作。深入研究国家和省上政策、规划，加强与国家和省上的衔接，积极做好项目申报、争取工作。围绕重大基础设施、新型工业、现代农业、文化旅游、市场体系建设等重点，谋划、优化、储备一批发展前景广阔的项目。注重优化投资结构，加大产业链项目、生活消费品工业的投资力度，促使产业体系不断完善。

加快项目建设进度。落实重点建设项目管理责任制，确保项目按照时间节点顺利推进。实施好火车站综合改造、中心广场北扩、水井巷商务区等重点项目，加快打造精品街区。做好西宁机场扩能改造二期、兰新铁路第二双线等项目协调服务工作，抓好2012年城洽会签约项目落地工作。同时，以高度的政治责任，全面完成玉树灾后援建任务，为玉树各族人民交一份满意的答卷。

　　加大招商引资力度。以"招商引资年"为抓手，把扩大民间投资作为重要着力点，落实国家和省上促进民间投资的政策，创新方式，创造公平、公正、法治的招商亲商氛围和环境。办好第二届"城洽会"，加强项目推介，努力提高项目的签约率和履约率，力争引进重大项目有新突破。

　　（二）优化结构转变方式，在提高质量效益上实现新突破

　　积极推进经济结构调整和发展方式转变，加快产业转型升级，追求实实在在、没有水分、惠及民生的发展，促进经济增长速度与结构、质量、效益相统一。

　　做强优势特色工业。紧紧围绕省上确定的十大特色优势产业，落实省市促进工业发展的各项政策措施，全力服务支持经济技术开发区。加快实施"双百"项目和重大技术进步项目，建设特色优势产业基地，力争在园区形成销售收入超千亿元的有色金属精深加工基地、销售收入超500亿元的特色化工和太阳能光伏制造基地、销售收入超百亿元的藏毯绒纺、高原生物制品、装备制造、轻金属材料、锂电材料及储能电池产业基地。推进企业技术进步，建设企业技术研发中心和国家级新能源高新技术产业化基地，加快发展循环经济，推进"两型"园区建设，在建设"国家循环经济发展先行区"上走在全省前列。大力扶持轻工业发展，优化工业结构。制定以区县为单位的全面建成小康社会的行动方案。进一步完善县域产业园区发展规划，加强基础设施建设，抓紧确定产业重点和布局，加大招商引资力度，争取项目落地实施，加快集聚发展，把县域工业园区做实做大做强。

　　繁荣活跃第三产业。把促进文化旅游体育融合发展作为长期战略，以建设高原山水花园旅游城市为目标，深入挖掘西宁文化旅游体育资源优势，抓好西宁旅游服务中心、青海自然博物馆、体育公园等项目建设。引导社会宾馆、低星级宾馆升级改造，继续扶持和规范小微宾馆，提升接待水平。改造提升传统服务业，规范提升专业市场，建设地方特色旅游产品等大型专业超市，运营好青藏高原农副产品集散中心。加快发展物流、金融、中介等现代服务业，建设青海（西宁）国际清真产业园、双寨物流园。调整专业市场布局，引导产业低端环节、生产环节向城市外围有序转移。积极开拓城乡消费市场，改善消费环境，强化消费拉动作用。整合文化、旅游、体育等专项资金，统筹推进文化、旅游等服务业态集聚发展，加快打造各具特色、不同定位的服务业集聚区。

　　培育壮大市场主体。把服务企业作为政府工作的重要内容和重要要求，创造更好的发展环境，加快培育市场主体"提标扩面"。大力发展非公有制经济，采取更加有效的措施，增强经济发展活力，使非公有制经济成为经济建设和社会发展的有生力量。继续完善政策，做好服务，加快区县创业孵化园建设。扶持中小微企业发展，扩大小额担保贷款支持额度。按照常态化、制度化的要求帮扶企业，让更多的小企业发展成为龙头企业、规模以上企业、科技型企业，力争年内新增小微企业2000家以上、规模以上工业企业40家以上。

（三）加快推进城镇化，在统筹城乡一体发展上实现新突破

　　把握国家加快推进城镇化的重大机遇，积极探索西宁集约、智能、绿色、低碳的新型城镇化道路，在统筹城乡发展上迈出新步伐。

　　加快推进小城镇建设。把县城和中心镇作为区域发展的龙头和关节点，强化中心带动作用。按照市区的标准规划建设县城，按照县城的标准规划建设小城镇，积极扶持基础条件好、发展潜力大的乡镇率先建设，如大通的城关、长宁，湟中的上新庄、甘河滩，湟源的日月、大华等，实施一批道路、供排水、垃圾污水处理等项目。结合农村新型社区建设，积极稳妥地推进人口较多、条件较好的村就地城镇化，逐步形成布局合理、功能完善、层次分明的城镇网络体系。

　　突出产业支撑作用。制定完善推动产业发展的政策，确定产业重点和布局，加大招商引资力度，争取项目落地建设，做到城镇化与产业园区建设、农业产业结构调整、农业产业化经营、发展非公经济有机结合，不断增强产业支撑能力，促进产城融合，以园区带动城区，努力实现"城乡一体、两区同建、四化同步"。

　　加快发展现代农业。加大"菜篮子"建设力度，新建和改造温室4500栋，力争蔬菜种植面积达到32万亩，产量达到82万吨，提高温棚冬季产出率。新建畜棚4000栋，建设良种奶牛基地、肉牛羊养殖基地、生猪养殖基地各10个，禽类基地5个，增强市场供应能力。抓好农业科技示范园区建设及城北区都市休闲观光农业园、城中区总寨高原型现代都市农业科技示范园、大通和湟中现代农业示范园、湟源现代畜牧业示范园建设。着力培育新型经营主体和服务主体，发展专业合作社。积极扶持农业产业化龙头企业，发挥示范带动作用。做强油菜籽、蚕豆、马铃薯等特色产品。加强绿色生产，从源头上确保农产品质量安全。

　　推进新农村建设。以"党政军企共建示范村活动"带动新农村建设。抓好灌区续建配套及节水改造、设施农业水利配套等工程，年内解决5万人、5万头（只）牲畜饮水安全问题，加强农村生活污水处理设施建设。继续强化扶贫开发工作，实施好70个村扶贫整村推进和15个易地搬迁项目。加大农民特别是失地农民的创业就业培训工作，增强就业能力，实现农村转移劳动力35万人次的目标。

（四）加强规划与建设，在提升城市功能上实现新突破

强化城市建设的财富积累意识，按照"旧城提升功能、新区完善繁荣、积极启动新城、统筹全域发展"的思路，加快城市规划建设"提标扩面"，在建设宜居之地、休闲之都上有新进步。

　　提升规划水平。加强东部城市群建设深层次研究，强化对重大基础设施和项目的谋划布局，提升西宁区域引领、服务、辐射功能。强化人脉、文脉、山脉、水脉等城市特色研究，丰富人文西宁新内涵。加强城市地下空间资源的规划研究，做到当前有效利用、长远不留遗憾。

　　推进旧城改造。抓紧实施建国路、新宁路、海湖路、黄河路等片区重点项目。做好城市核心区地标性建筑的前期启动工作。加快南绕城高速公路等环西宁高等级路网建设，加强西宁主城区与东部城市群的主干路衔接，强化市区和重点镇、副中心城市之间交通环的构建。完成主干道大修整治，建成海山路、三其西路等工程，开工建设门源路、博文路南延段等项目，改造南川河洪水桥。抓好"畅通交通"工程，继续建设一批过街天桥，打通一批断头路，加大退市还路力度，开辟更多街巷小循环，增设部分路口立交，提升交通指挥和公交车辆调度智能化水平。继续优化快速公交、直达公交线路和站点设置，加快市中心停车场提升改造。筹资更新达到报废期限的公交车辆。科学规划人行道建设，对反复开挖、重复建设现象追究责任。完善城市配电网、天然气门站、热力管网建设，加大供排水管网改造力度，不断提高市政功能设施的保障能力。

　　加快新城建设。不断完善海湖新区配套，推动万达城市综合体、海湖新区医院等项目建设。深化对多巴城市副中心的定位和发展规划的研究，积极推动多巴新城建设。依托海湖新区、南川工业园、甘河工业园以及将启动建设的鲁沙尔旅游区、多巴新区、西川新城等，抓紧做好西宁新区规划建设的各项前期工作，年内启动建设。

加大拆迁力度。建立完善拆迁征地工作新机制，加强正面宣传和正确引导，依法依规推进拆迁工作，维护群众合法利益，有效保障重点项目顺利实施。进一步加大拆除违法违章建筑工作力度，坚决遏制乱搭乱建现象。通过征地拆迁，使我们的城市建设更快、发展更好、人民生活更加幸福！

（五）坚持以人为本理念，在改善社会民生上实现新突破

顺应各族人民对美好生活的新向往，按照"守住底线、突出重点、完善制度、引导舆论"的要求，量力而行、尽力而为，加快民生改善"提标扩面"，努力让人民过上更好生活。

　　按照"学有所教"的要求，办好人民满意的教育。加快普及学前教育和高中阶段教育，认真落实省上"1+9+3"教育保障机制，实现教育投资11.8亿元，实施41所标准化学校建设和农村初中建设工程。加快一、二期布局调整工程进度。加强学前教育办学能力，新建11所农村幼儿园，建设、改造7所城镇幼儿园。实施世纪职校等5个中等职业学校建设工程，着力提高职业教育办学竞争力。积极引进民间资本和办学品牌，促进教育多元化发展。加强教师队伍建设，把立德树人作为教育工作的根本任务，推进教育内涵式发展，让每个孩子都成为有用之才。

　　按照"劳有所得"的要求，实施就业优先战略。统筹产业转型升级、企业培育壮大和就业优质充分三者关系，致力创造财富。加强创业孵化基地建设和技能培训，重点实施面向尚未就业的高校和中专毕业生专项就业培训，不断提高适业人群就业的素质和能力。加大劳务经纪人队伍培训，落实农民工培训计划，切实提高劳动力就业创业能力，力争年内新增就业3万人以上。积极创造良好的环境，支持民众自主创业。积极引导群众艰苦创业、勤劳致富，用自己的双手创造美好生活！

　　按照"病有所医"的要求，提升公共卫生服务水平。加大卫生建设项目实施力度，开工建设市一医院门诊综合楼、湟中和湟源县急救中心及三县乡镇卫生院改扩建项目。加强县级医院能力建设。加快城市社区卫生服务中心（站）规范化建设，为群众提供安全、有效、方便、价廉的医疗和公共卫生服务。积极推进城乡居民大病保险商业化运作。启动"健康西宁"创建活动，全面推进县级公立医院改革，创建医患和谐示范区。推进国家免费孕前优生健康检查项目，多层次为育龄人群提供计划生育优质服务。

　　按照"老有所养"的要求，完善社会保障工作。尊重和关爱城乡弱势群体。高度重视老龄工作，加快推进社区日间照料中心和农村老年人综合服务中心等建设。强化"全覆盖、多层次、保基本、可持续"的社会保障体系建设，积极推进城乡居民大病保险商业化运作，探索城镇职工医保大病保险商业化运作工作。推进居民社保城乡一体化，逐步缩小城乡居民社会养老保险缴费和待遇差距。积极推进社会救助与社会保险、扶贫开发、慈善事业、劳动就业的有效对接，努力形成配套衔接的社会救助机制。

　　按照"住有所居"的要求，扎实推进安居工程。继续加强城镇各类保障性住房建设和管理，加快棚户区改造，强化公租房建设的针对性。年内建设各类城镇保障性住房36789套、农村危房改造7000户、农村奖励性住房17000户。坚持把全面提升各类小区物业服务水平作为百姓幸福安宁的一件大事来抓，实现居住小区物业管理全覆盖。

　　按照"文化西宁"的要求，提升城市软实力。扎实推进文化惠民工程，统筹谋划市图书馆、档案馆、文化传媒大厦等场馆建设。启动"街区24小时自助图书馆"项目。全力打造特色文化品牌，注重挖掘和开发河湟文化、昆仑文化等传统文化，集中打造1——2部有市场影响力、具有西宁特色的优秀精品剧目。开发民族文化、藏医药文化等产业，抓好西宁文化创意产业园、民族演艺厅等项目建设。保护利用好山陕会馆、西宁城隍庙等文物古迹，规划建设塔尔寺大景区。加大公共体育设施投入，进一步活跃群众性体育活动。以创建全国文明城市为载体，弘扬社会主义核心价值观和新青海精神及西宁精神，为经济社会发展提供健康持久的动力。

　　按照"创新驱动"的要求，增强科技支撑作用。围绕国家创新型试点城市目标，实施科技重大专项，提升企业核心竞争力和自主创新能力，着力构建以企业为主体、市场为导向、产学研相结合的技术创新体系。引导新能源、新材料关键技术、节能减排技术，生物资源开发等研究，加强农业科技成果推广应用，加快建设产学研创新基地，不断增强创新能力。

　　按照"多措并举"的要求，确保物价基本稳定。建基地、促流通、管市场多措并举，加大直销体系建设力度，降低流通成本。开展肉类蔬菜追溯体系试点城市创建，不断提升质量安全和供应保障水平。进一步完善价格异常波动应急处理机制，继续落实好价格补贴等政策，加强价格干预，严厉查处和打击价格违法行为，力争实现调控目标。

　　与此同时，我们还将继续实施一批为民办实事项目，竭尽全力干群众最需要和最认可的事，最大限度满足群众需求，努力让人民群众的幸福指数有新的提升！

　　（六）着力改善生态环境，在建设美丽家园上实现新突破

　　把"美丽西宁"作为美丽中国建设的重要组成部分，推进"国家森林城市"创建步伐，在建设"国家生态文明发展先行区"上走在全省前列。

　　加快城市绿网建设。继续推进大南山生态绿色屏障、城市绿地、主题公园和休闲场地建设及北山危岩体综合治理。完成天然林、三北防护林等重点工程29.1万亩，继续提高森林覆盖率。加快城市出口道路绿化美化和城区"三河六岸"绿色滨河景观带建设，重视市花栽种，实现山边、路边、河边、田边绿量大幅增加，努力构建以城区为核心、南北两山为屏障、县域为纵深的三环生态布局。加强农村生态建设、环境保护和综合整治，努力建设美丽乡村。

　　优化城市水网建设。加强对市域各流域水系保护和综合开发利用，统筹城市群水资源优化配置，加快重点水源及水源涵养工程建设，实施第七水厂扩建，全面提升供水质量，扩大供水覆盖面。积极谋划战略性调水工程，支持引大济湟工程建设，新建东部城市群东区供水工程。强化以治政治污为主的湟水流域（西宁段）水污染防治工作，实现南川河清水入城。抓好北川河综合治理，实施宁湖及周边河道治理，建设滨水公园。推进湟水流域由单一治污向集污染防治、生态建设、景观休闲和防洪泄洪为一体的综合治理转变。抓好市域内灌渠的开发利用，增加水面和景致，为市民提供更多休闲去处。

　　深入推进创模工作。按照控制污染物总量、改善环境质量、防范环境风险的总体要求，做好创建国家环保模范城市省级预评估验收工作。加大环境保护监管力度，加快建设大通煤矿地质环境治理示范工程。加强工业污染和重金属污染防治，实施环保重点工程，制定主要污染物总量控制和减排计划，做好火电厂脱硫脱硝、水泥厂脱硝等重点减排工程项目，完成"两废"中心建设。巩固排污口整治成果，实施排水管网改造工作，加快雨污分流，加大污水收集量。开展大气综合治理，加强细颗粒物（PM2.5）监测工作，力争空气质量优良率保持在85%以上。加大噪音污染、光污染防治力度，给群众一个清静的生活环境。对各类项目严把环保关，不符合环保要求的项目一概不许引进、不得实施！

　　（七）深化体制机制改革，在强化精细管理上实现新突破

　　深化改革不能犹豫观望，不能迟疑不前，不能无所作为，必须在解放思想中解放生产力，在更大程度、更大范围内发挥市场配置资源的基础性作用。全面落实中央和省委、省政府安排部署的各项改革措施，进一步增强加快发展的内在动力和活力。

　　深化行政体制改革。深入推进政企分开、政资分开、政事分开、政社分开，加快转变政府职能。把应该由市场和社会发挥作用的交给市场和社会，政府切实承担起创造良好环境、提供公共服务、维护社会公平的职责。争取、创新、整合各类生产要素。建立健全公务员激励约束机制和考核考评办法。进一步明确市、县（区）两级权责，释放县、区发展潜力。深化国有资产管理体制改革，创新行政事业单位资产管理体制，盘活市级行政事业单位资产，实行产权集中管理、经营，增加政府可用财力，提升资产管理效益。试行国有资本经营预算制度，促进国有资本收益的合理配置和使用。

深化教育文化卫生体制改革。探索理顺义务教育区划管理体制，推进考试和招生制度改革，完善义务教育免试相对就近入学的具体办法。实现西宁广播电视台制播分离，深化文化团体改革，使其成为精品剧目生产基地和文化演艺市场生力军。深化医药卫生体制改革，完善补偿机制，落实医院自主经营管理。

深化投融资体制改革。充分利用银行贷款、发行票据、信托、BOT等方式，力争融资突破120亿元，加快城投公司三期企业债券发行工作。建立健全多层次创业投资和中小企业贷款担保体系，缓解中小企业融资困难。建立市区联动的土地储备机制，加快土地一级开发，进一步盘活城市资源。

加强社会管理创新。充分发挥城乡社区、企事业单位、人民团体、社会组织的作用，发挥群众的基础作用，推广"3+6"模式。进一步加强社区建设与管理，健全完善以社区为基础的服务体系，探索实践社区工作专业化、社区干部职业化的新路子，切实提高基层服务群众的能力和水平。建设"法治西宁"，运用法治思维和法治方式深化改革、推动发展、化解矛盾、维护稳定。继续加强安全生产，规划规范一批应急避险场所。强化社会治安综合治理，严厉打击各类刑事犯罪和邪教活动，全力做好涉藏维稳工作，在建设"民族团结进步示范区"上走在全省前列，让全市各族人民生活得更加平安、更加祥和！

　　深化户籍制度改革。把有序推进农业转移人口市民化作为一项重要任务，加快城中村综合改造和园区村庄搬迁，加快县域城镇化步伐，在社会保障、医疗卫生、教育文化、就业技能培训等方面完善制度措施，推进公共服务均等化。

深化公交体制改革。深化城市公交管理体制和经营机制改革，增强城市公交经营活力和可持续发展能力，不断提高服务水平，提升市民出行便捷程度，努力打造人民满意的城市公交！

三、加强政府自身建设

　　我们的政府是为人民服务、对人民负责、受人民监督的政府。我们将顺应人民的要求，致力建设为民、务实、高效、廉洁的服务型、学习型、创新型政府。

　　切实践行执政为民宗旨，致力建设服务政府。坚持把群众满意不满意、高兴不高兴、答应不答应作为衡量政府工作的唯一标准，体察感受群众疾苦，倾听顺应群众呼声，与人民群众同苦、同乐、同干。建立健全联系群众、服务群众的制度机制，始终保持同人民群众的血肉联系，努力实现好、维护好、发展好最广大人民的根本利益！

　　严格依法行政接受监督，致力建设法治政府。加强政府立法，严格规范性文件管理，落实行政执法责任制，促进行政行为规范有序。自觉接受人大法律监督、政协民主监督和社会公众及舆论监督，认真办理建议和提案。坚持科学民主决策，推进政务公开，保障人民群众的知情权、参与权、表达权、监督权。

　　切实改进作风强化执行，致力建设效能政府。坚持"实干富民强市"，强化政府公信力和执行力，坚决落实中央八项规定，少开会、开短会、讲实话，腾出更多的时间搞调研、谋工作、抓落实，省出更多的经费办实事、惠民生。加强学习、注重研究，推行我市拆迁工作中形成的现场工作法，严格落实工作限时办结制、问效追责制，以创新的思路、创新的方式、创新的成效开展工作，坚决克服不思进取、取巧偷懒、平庸守成的现象！

加强勤政廉政夯实基础，致力建设廉洁政府。以勤政提升干部，以廉政保护干部。全面落实党风廉政建设责任"一票否决制"，深入推进"一岗双责"和廉政风险防控管理工作。转作风，提效率，开展"向人民汇报、让人民评议"现场直播活动。加强公益项目中廉政文化设施建设工作。强化行政监察和审计监督，加大重点领域、关键环节专项治理力度，严肃查办违纪违法案件。坚决反对铺张浪费，力戒奢靡之风，做到干部清正、政府清廉、政治清明。

各位代表，政府工作，责任如山！让我们紧密团结在以习近平同志为总书记的党中央周围，在省委、省政府和市委的坚强领导下，进一步解放思想，坚定信心，开拓创新，砥砺前行，为全面建成小康社会而努力奋斗!
